# Social media and adolescent mental health: A consensus report of the National Academies of Sciences, Engineering, and Medicine

**DOI:** 10.1093/pnasnexus/pgae037

**Published:** 2024-02-27

**Authors:** Sandro Galea, Gillian J Buckley

**Affiliations:** Robert A Knox Professor, School of Public Health, Boston University, Boston, MA 02118, USA; Senior Program Officer, National Academies of Sciences, Engineering, and Medicine, Washington, DC, USA

A range of digitally mediated communication platforms—loosely called as a group, social media—have transformed how the world communicates and interacts over the past decade and a half. The rise in popularity of some social media platforms has been nothing short of extraordinary. For example, Facebook, introduced in 2004, now has 2.9 billion regular users (1, 2). Snapchat has 397 million regular users, and TikTok, introduced only in 2018, has 1.7 billion regular users (2). As with all new technologies, the most common users of new social media platforms have been young people, with adolescents being the fastest adopters of these technologies and their most avid users.

At the same time as the world has witnessed this rise in social media use, the US has seen an increase in mental disorders overall, and among adolescents in particular. Rates of mental health problems have increased over the past decade, as have suicidal ideation and completed suicides in this group (3, 4). This has led, not unreasonably, to concern that this worsening in adolescent mental health is linked to the rise in social media use. This thesis has been proposed in academic writing (5, 6), featured in a number of prominent books and articles for a broader public audience (7–10). Concern about this connection was fueled by the release of documents from Facebook by a whistleblower that showed both that social media platforms were aware of the risks of worsening mental health and that these same companies made concerted efforts to ensure continued and ever greater engagement with their platforms (11–13).

With this potential link in mind, the National Academies of Sciences, Engineering, and Medicine convened a consensus committee with a charge to disentangle the links between social media use and adolescent mental health and to articulate recommendations accordingly. The committee's Statement of Task and membership are provided in Appendixes A and B. This work culminated in a report that was just released (14). We summarize here both the report's main findings and the recommendations that emerged from the committee's deliberations.

## The link between social media and adolescent mental health

The question that has animated such public discussion and debate—the potential link between social media and adolescent mental health—turns out to be far more complex than might appear at first glance, and there is no easy answer to whether increasing social media use is associated with growing mental distress for adolescents. There are three principal reasons for the challenge in answering this question.

First, the term social media is broad, and there is no easy catchall that can describe the range of interactive digital tools that are popularly called “social media.” For the purposes of its deliberation, the committee relied on a definition adapted from the American Psychological Association, referring to social media as the set of “interactive technologies that facilitate the creation and sharing of information, ideas, interests, and other forms of expression through virtual communities and networks” (15). This definition encompasses a broad range of tools, and each of these tools operate in different ways. Importantly, the notion of use of social media encompasses a range of behaviors that are irreducible to simple reductions (use vs. nonuse) and must be understood as a heterogenous set of exposures. For example, simply focusing on length of time that adolescents use a particular social media platform does not account for whether that adolescent is using that time to stream movies in their spare time, to connect to communities of peers who may share interests productively, or to engage in exchanges that may expose adolescents to risk of exacerbating negative body image. Therefore, the question posed at the outset is virtually unanswerable without a careful assessment of the types of engagements adolescents have with social media and linking those engagements to specific mental health indicators.

Second, and relatedly, the literature is quite far from being able to provide confidence that particular behaviors—with the exception of some explicitly harmful engagements as noted below—are linked to adverse mental health. This challenge emerges from a limited literature, characterized by a paucity of longitudinal studies, and by, in the main, use of crude exposure measures that fail to differentiate between specific behaviors while adolescents engage with social media. This suggests that much more work is needed to appropriately determine which behaviors, when, and by whom might be associated with mental health harms among adolescents.

Third, while this report was catalyzed by concern about the effect of social media on adverse mental health, it is apparent that entertainment, giving pleasure, and providing connections are important benefits of social media, particularly for isolated adolescents (e.g. adolescents with disabilities, or LGBTQ+ adolescents living in rural areas). Hence, in the big picture, any assessment of overall harms—and efforts to restrict social media use commensurately—must be balanced with the clear benefits that many users of social media derive from engagement with these platforms.

It is however apparent that some behaviors—even if rare—are explicitly harmful and emerge from social media exposure by adolescents. These behaviors, including cyber-stalking and harassment, are well documented and most troubling because of the difficulty that social media has in policing these behaviors—or even in providing opportunities for adolescents who experience them to report these behaviors or shield themselves from them.

## Recommendations for a better social media use

The committee developed a set of recommendations that aim to help better understand the question at hand, deal with immediate challenges, and establish the foundations for healthier social media use going forward.

First, the committee noted the need for much more research in the area. It is astonishing that social media have become so ubiquitous without any careful consideration about its potential impact on health, particularly the health of adolescents who are deeply influenced by such exposure at a formative stage in their life. By way of analogy, it is as though a brand-new food group were released into the public, that essentially everyone consumes every day, without any investigation into its safety. This therefore calls for the need for much more research in the area, particularly longitudinal research that allows for the measurement of specificity of exposure so that we can discern which elements of social media use are harmful and which are not. In parallel, the committee recommended that the International Organization for Standardization should convene an ongoing technical working group that includes industry representatives, civil society, and academic stakeholders to develop standards for social media platform design, transparency, and data use that can, through emerging data, ensure best practices in social media platforms toward mitigating potential harms.

Second, the committee felt strongly that enough is known about the harms of cyber-harassment that efforts do need to be expended to mitigate this harm. This includes the development of systems for reporting, follow-up, and adjudication for cases of online harassment and abuse. These systems should be easy to use, universal, accountable, and transparent. In addition, the committee suggested that the US Substance Abuse and Mental Health Services Administration should have intervention programs for children and adolescents who experience digital abuse. This would be a step toward creating an infrastructure of support for an experience that is becoming distressingly common and casting a pall on social media use that may, in the main, be otherwise benign.

Third, given the ubiquity of social media use, it is somewhat remarkable that the country has not engaged in any formal efforts to teach safe, healthy, social media use by adolescents. This can only be done in the schools, and the committee recognized that as such teachers need to be prepared to engage in teaching in this area. This will require that state boards of education set standards for comprehensive digital media literacy education in grades K through 12. In addition, the Council for the Accreditation of Educator Preparation would need to set requirements for media literacy education for student teachers and as part of ongoing professional development for veteran teachers. An extension of this includes the training of medical providers—through relevant bodies—in understanding social media and its consequences, so that clinical providers are in a position to counsel patients on social media use and spot potential warning signs.

## Conclusion

Informed by the rapid spread of social media, and the co-occurring crisis in adolescent mental health, the National Academies of Sciences, Engineering, and Medicine consensus committee was tasked with addressing whether the former is leading to the latter. The answer turns out to be nowhere near as simple as we might hope. The question does, however, prompt the need for careful research in the area, elevate the importance of short-term tackling of harassment through social media, and encourage the establishment of pathways to ensure teaching about social media in schools and counseling about social media use by medical providers.

## Supplementary Material

pgae037_Supplementary_Data

## References

[1] Rothman L . 2015 Feb 24. Happy birthday, Facebook. Time. Accessed 2023 Jul 11. https://time.com/3686124/happy-birthday-facebook.

[2] World Population Review. Facebook users by country 2023. Accessed 2023 Jul 11. https://worldpopulationreview.com/country-rankings/facebook-users-by-country.

[3] Center for Disease Control and Prevention . 2023. Youth risk behavior survey: data summary and trends report 2011–2021. Accessed 2023 July 11. https://www.cdc.gov/healthyyouth/data/yrbs/pdf/YRBS_Data-Summary-Trends_Report2023_508.pdf.

[4] Joseph VA , et al 2022. Temporal trends in suicide methods among adolescents in the US. JAMA Network Open. 5(10):e2236049–e2236049. 36223121 10.1001/jamanetworkopen.2022.36049 PMC9557855

[5] Twenge JM , Haidt J, Lozano J, Cummins KM. 2022. Specification curve analysis shows that social media use is linked to poor mental health, especially among girls. Acta Psychol (Amst). 224:103512. 35101738 10.1016/j.actpsy.2022.103512

[6] Orben A , Przybylski AK. 2019. Screens, teens, and psychological well-being: evidence from three time-use-diary studies. Psychol Sci. 30(5):682–696. 30939250 10.1177/0956797619830329 PMC6512056

[7] Pearson C . 2023 May 23. Does your child have an unhealthy relationship to social media? Here’s how to tell. New York Times. Accessed 2023 Jul 11. https://www.nytimes.com/2023/05/23/well/family/social-media-use-children-parents.html.

[8] Joyce A . 2023 May 5. Limiting teens’ social media feels impossible. But we have to try. Washington Post. Accessed 2023 Jul 11. https://www.washingtonpost.com/parenting/2023/05/25/social-media-mental-health-parenting/.

[9] Twenge JM . 2019. Igen: why today's super-connected kids are growing up less rebellious, more tolerant, less happy—and completely unprepared for adulthood—and what that means for the rest of us. Atria Books.

[10] Heitner D . 2023. Growing up in public: coming of age in a digital world. TarcherPerigee.

[11] Wall Street Journal . 2021 Sep 29. Teen mental health deep dive. https://s.wsj.net/public/resources/documents/teen-mental-health-deep-dive.pdf.

[12] Horwitz J . 2021 Oct 3. The Facebook whistleblower, Frances Haugen, says she wants to fix the company, not harm it. Accessed March 2023. https://www.wsj.com/articles/facebook-whistleblower-frances-haugen-says-she-wants-to-fix-the-company-not-harm-it-11633304122.

[13] Wells G , Seetharaman D, Horwitz J. 2021 Nov 5. Is Facebook bad for you? It is for about 360 million users, company survey suggests. Wall Street Journal. Accessed 2023 Mar. https://www.wsj.com/articles/facebook-bad-for-you-360-million-users-say-yes-company-documents-facebook-files-11636124681.

[14] National Academies of Sciences, Engineering, and Medicine . 2023. Social media and adolescent health. Washington, DC: The National Academies Press. 10.17226/27396. 38713784

[15] American Psychological Association . Social media and the internet. Accessed 2023 Jul 11. https://www.apa.org/topics/social-media-internet.

