# Supplementary material for: Social media and adolescent mental health: A consensus report of the National Academies of Sciences, Engineering, and Medicine
Source: PNAS Nexus. 2024 Feb 27;3(2):pgae037. doi: 10.1093/pnasnexus/pgae037 (PMC10898856; doi:10.1093/pnasnexus/pgae037)
Supplement: pgae037_Supplementary_Data [file pgae037_supplementary_data.pdf]

Appendix A: Statement of Task

An ad hoc committee of the National Academies of Sciences, Engineering, and Medicine will examine the current research and make conclusions about the impact of social media on the mental and physical health and wellbeing of adolescents and children. The committee will consider the following questions:

1. In what ways, if any, does social/digital media affect the mental and physical health and wellbeing of adolescents and children (age 13 -18 yrs), including anxiety, depression, addiction and self-efficacy, social isolation, relationship malformation, relationship with their parents, life satisfaction and physical activity?

   a. Do these effects differ between different social/digital media use (e.g. social media vs video streamers)?

   b. Do the effects of social/digital media on adolescents and children differ between different demographics of children (race and ethnicity, gender, socio-economic status)?

2. In what ways, if any, does the product design of social media (e.g., consumer retention strategies, data profiling, advertising, and others) affect adolescents and children's physical and mental health and wellbeing?

3. What consequences, if any, do the effects of social/digital media on adolescents and children's mental and physical health and wellbeing have for education, social development, family dynamics, and projected economic prospects?

4. Do new forms of social media (such as 3D social networking) raise novel questions for the health and wellbeing of adolescents and children and their families?

The committee should identify what is needed in a research agenda to more fully understand the impact of social media on adolescents, children and their families, as well as the data that would be required in order to comprehensively evaluate the effects of social media products on the mental and physical health and wellbeing of adolescents and children.

The committee should also make recommendations for steps that parents, social media companies, and public officials can take to maximize potential benefits and minimize potential harms of social media for adolescents and children.

Appendix B: Committee Members

Sandro Galea, MD, DrPH. (Chair)
Dean
Robert Knox Professor, School of Public Health
Boston University School of Public Health

Ceren Budak, PhD
Associate Professor of Information, School of Information
Associate Professor of Electrical Engineering and Computer Science, College of Engineering
Faculty Associate, Center for Political Studies, Institute for Social Research
University of Michigan

Munmun De Choudhury, PhD
Associate Professor
Director, Social Dynamics and Wellbeing Lab
Georgia Tech University

Douglas Gentile, PhD, MA
Professor of Psychology
Iowa State University

Amanda Guyer, PhD, MPhil, MS
Assoiate Director, Center for Mind and Brain
Professor, Human Development and Family Studies
University of California, Davis

Jeff Hancock, PhD
Professor of Communication
Founding Director, Stanford Social Media Lab
Stanford University

Julianne Holt-Lunstad, PhD
Professor of Psychology and Neuroscience
Brigham Young University

Stephanie Reich, PhD
Professor, Education
University of California, Irvine

Miguel Sarzosa, PhD, MSc
Assistant Professor of Economics
Purdue University Krannert School of Management

Leslie Walker-Harding, MD
Division Chief and Director
Ford/Morgan Endowed Professor and Chair
Seattle Children's Hospital
Department of Pediatrics
University of Washington

Christopher Yoo, JD, MBA
John H. Chestnut Professr of Law, Communication, and Computer and Information Science

Founding Director, Center for Technology, Innovation, and Competition
University of Pennsylvania Penn Carey Law
